# Diclofenac, ibuprofen, and paracetamol biodegradation: overconsumed non-steroidal anti-inflammatories drugs at COVID-19 pandemic

**DOI:** 10.3389/fmicb.2023.1207664

**Published:** 2023-10-30

**Authors:** Beatriz L. Ferreira, Dionisia P. Ferreira, Swanny F. Borges, Adriana M. Ferreira, Fabricio H. Holanda, João G. M. Ucella-Filho, Rodrigo Alves S. Cruz, Willian G. Birolli, Rafael Luque, Irlon M. Ferreira

**Affiliations:** ^1^Biocatalysis and Applied Organic Synthesis Laboratory, Federal University of Amapá, Macapá, AP, Brazil; ^2^Department of Forestry and Wood Sciences, Federal University of Espírito Santo, Jerônimo Monteiro, Espirito Santo, Brazil; ^3^Molecular Oncology Research Center, Institute of Learning and Research, Barretos Cancer Hospital, Barretos, SP, Brazil; ^4^Universidad ECOTEC, Via Principal Campus Ecotec, Samborondón, Ecuador

**Keywords:** COVID-19, mycodegradation, contaminants, SARS-CoV-2, NSAIDs

## Abstract

The consumption of non-steroidal anti-inflammatory drugs (NSAIDs) have increased significantly in the last years (2020–2022), especially for patients in COVID-19 treatment. NSAIDs such as diclofenac, ibuprofen, and paracetamol are often available without restrictions, being employed without medical supervision for basic symptoms of inflammatory processes. Furthermore, these compounds are increasingly present in nature constituting complex mixtures discarded at domestic and hospital sewage/wastewater. Therefore, this review emphasizes the biodegradation of diclofenac, ibuprofen, and paracetamol by pure cultures or consortia of fungi and bacteria at *in vitro*, *in situ*, and *ex situ* processes. Considering the influence of different factors (inoculum dose, pH, temperature, co-factors, reaction time, and microbial isolation medium) relevant for the identification of highly efficient alternatives for pharmaceuticals decontamination, since biologically active micropollutants became a worldwide issue that should be carefully addressed. In addition, we present a quantitative bibliometric survey, which reinforces that the consumption of these drugs and consequently their impact on the environment goes beyond the epidemiological control of COVID-19.

## 1. Introduction

In a turnaround moment for humanity, 27 cases of pneumonia of unknown etiology were reported on 31 December 2019 in the city of Wuhan, Hubei Province, China. Later, the fast-spreading agent was identified and named as Severe Acute Respiratory Syndrome Coronavirus 2 (SARS-CoV-2) by the International Committee on Taxonomy of Viruses (ICTV), and the new disease was known as Coronavirus Disease 2019 (COVID-19) by the World Health Organization (WHO) (Oscanoa et al., 2020; Hu et al., 2021).

In March 2020, WHO warned governments to constitute a response and a containment initiative to an epidemic, later classified as a pandemic scenario. A series of measures were proposed and used in an attempt to contain the virus spreading, including home quarantine of infected people and those who entered in contact with them (Zhang et al., 2021). However, the available tools and resources were not enough to prevent this threat to mankind.

Mild symptoms of SARS-CoV-2, as fever, shortness of breath, and diarrhea, can be misunderstood with different viruses. Whereas severe cases can lead to pneumonia, severe acute respiratory syndrome, renal failure, multiple organ failure, and death (Singhal, 2020). Although the pathogenesis of this disease is still not completely understood, growing evidence indicate that a dysregulated inflammatory syndrome is narrowly associated with COVID-19 severity and poor prognosis (Zoulikha et al., 2022), which results from the high levels of inflammatory cytokines like IL2, IL7, IL10, GCSF, IP10, MCP1, MIP1A, and TNFα (Chen et al., 2020).

During the initial pandemic period, different known anti-viral, anti-malarial, and anti-inflammatory drugs were explored and introduced into the treatment. In this context, non-steroidal anti-inflammatory drugs (NSAIDs) were widely employed for COVID-19, especially for patients in home care. With over-the-counter availability, they have often been used without medical supervision to treat the basic symptoms of COVID-19, promoting excessive NSAIDs detection in wastewater treatment plants at both domestic and hospital sewage (Barcelo, 2020). Also, it is important to note that the consumption of these drugs increased significantly during the COVID-19 pandemic, even in comparison to other types of medication (Luis López-Miranda et al., 2022).

Before this period, these compounds were already among the most consumed drugs considering both with and without a prescription (Osafo et al., 2017). Therefore, it is important to note that the continuous use, even at sub-therapeutic concentrations, represents a potential risk to public health, although it is not possible to evaluate the effects of exposure at present (Stackelberg et al., 2004; Santos et al., 2010).

It is known that many non-target organisms (which possess human-and animal-alike metabolic pathways, similar receptors, or biomolecules) are inadvertently exposed to these active pharmaceuticals released into the environment (Fent et al., 2006; Santos et al., 2010). A problem that can be aggravated by the constitution of complex mixtures of biological products that, in some cases, can originate unknown super contaminants (Fent et al., 2006). Recently, the metabolic excretion of NSAIDs had received attention and became a relevant issue due to the impacts in aquatic species, more specifically in terms of chronic effects since low and constant contamination by these products were described (Banerjee and Maric, 2023). However, there is a large group of organisms whose exposure to these compounds and its effects have not yet been evaluated (Świacka et al., 2020).

Moreover, the removal of different NSAIDs through treatment stations has been reported as inefficient (Tiwari et al., 2017; Khasawneh and Palaniandy, 2021). Thus several strategies, including mycoremediation (Aracagök et al., 2018), bacterial remediation (Wojcieszyńska et al., 2022), membranes, and hybrid materials have been investigated for these purposes (Gu et al., 2018).

Based in the increased consumption of NSAIDs, especially by patients in COVID-19 treatment, these compounds were extensively detected in nature, as well as in domestic and hospital sewage/wastewater. Therefore, this review emphasizes the biodegradation of diclofenac, ibuprofen, and paracetamol, which are NSAIDs, by pure cultures and consortia of fungi or bacteria at *in vitro*, *in situ,* and *ex situ* processes over the past 10 years, as an efficient alternative for decontamination of these pharmaceuticals.

## 2. Pharmacology of the inflammatory process and possible COVID-19 impacts

The inflammatory process is considered a natural reaction of the organism in a protective response against harmful endogenous (immunological, neurological, and damaged cells) and exogenous effects (physical, chemical, and infectious), which involves different types of immune cells, blood vessels, chemical and molecular mediators. This process aims to block, inactivate, or eliminate harmful agents (Borges et al., 2018; Feehan and Gilroy, 2019). In most cases, the inflammation resolution occurs and the body returns to homeostasis after elimination. However, persistence of inflammatory cause and inefficient resolution can culminate in an exacerbated and prolonged inflammatory response that plays a key role in the development of multiple diseases (Badri et al., 2016; Feehan and Gilroy, 2019; Holanda et al., 2023).

The conventional pharmacologic intervention for the resolution of these inflammatory processes contemplate the use of anti-inflammatory drugs, which include the NSAIDs approached in this discussion, steroidal anti-inflammatory drugs (AIES) and/or glycocorticoids (Peesa et al., 2016).

Non-steroidal anti-inflammatory drugs present different chemical structures with similarities in the action mechanism, resulting in anti-inflammatory, antipyretic, and analgesic properties. These compounds are mainly indicated to treat symptoms of acute disorders (fever, edema, and pain), as well as in the treatment of chronic diseases. The main representatives of these drugs are diclofenac (DCF), ibuprofen (IBU), paracetamol (PAR), nimesulide, salicylic acid, piroxicam, meloxicam, naproxen, ketoprofen, mefenamic acid, and celecoxib (Chen et al., 2013; Wojcieszyńska et al., 2022).

**Figure 1:**
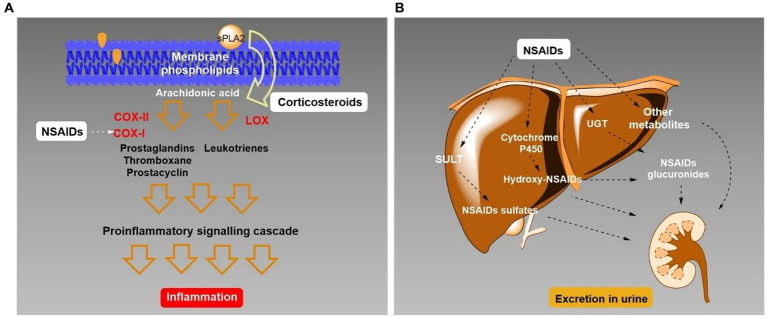
**(A)** Illustration of the simplified mechanism of action of nonsteroidal anti-inflammatory drugs (NSAIDs) in the inhibition of cyclooxygenase II. **(B)** Biotransformation route of NSAIDs in the liver.

Another active ingredient with increased consumption due to COVID-19 pandemic was paracetamol (PAR), also known as acetaminophen, which does not belong to the NSAIDs group because this compound does not present anti-inflammatory activity, but promotes analgesic and antipyretic properties similar to NSAIDs. Regardless of this, some studies have reported that PAR activity is also related to the inhibition of the cyclooxygenase (COX) enzymes, demonstrating a preferential inhibition of COX-2 (Ward and Alexander-Williams, 1999; Huntjens et al., 2005; Wojcieszyńska et al., 2022; Figures 1A,B).

The inflammatory process is considered an important part of the pathogenesis of various diseases (e.g., rheumatoid arthritis, diabetes, and cancer), and most of the time, it is a determinant factor regarding severity and complexity (Feehan and Gilroy, 2019). In this sense, several studies have already shown that, in the severe cases of COVID-19, the SARS-COV-2 virus infection promotes multisystem inflammation, generating intense tissue and cellular damage with respiratory, cardiovascular, liver, and renal complications concomitant with coagulation. Thus, demonstrating a strong relationship between disease aggravation and hyperinflammatory response (de Almeida et al., 2020; Micallef et al., 2020; Perico et al., 2023). Therefore, the clinical manifestations of COVID-19 can be a result from exacerbated inflammatory conditions for a considerable number of cases, leading to the use of anti-inflammatory drugs as therapeutic strategy.

## 3. Ecotoxicity of non-steroidal anti-inflammatory drugs

Pharmaceutical products can be released into the environment in different manners during their life cycle, including steps of production, distribution, acquisition, and home use. The three main routes to transform drugs into micropollutants are excretion after ingestion, medicine or chemical removal during bathing, and direct disposal of unwanted products due to leftovers or expired validity (Ruhoy and Daughton, 2007). In addition, topical medications can contribute significantly with the environmental contamination by IBU and DCF in relation to the amount released by patients employing orally taken products (Austin et al., 2021). For instance, DCF and IBU were determined in wastewater from treatment plants (WWTPs) and effluent receiving waters in Nigeria. Maximum concentrations in wastewater were 166.1 μg L^−1^ DCF and 62.0 μg.L^−1^ IBU (Ajibola et al., 2021). In addition, these authors also demonstrated a high environmental risk of IBU to fish and DCF to bacteria.

In another study, DCF and PAR were also determined in rivers in South Africa in the range of 40 ng L^−1^ to 51.94 μg L^−1^ for DCF and from 96.70 to almost 152 μg L^−1^ for PAR (Omotola and Olatunji, 2020). Furthermore, ketoprofen (42–133 ng L^−1^), IBU (73–126 ng L^−1^), DCF (199–469 ng L^−1^), mefenamic acid (8–13 ng L^−1^), and salicylic acid (54–109 ng L^−1^) were detected in the Besós river at urbanized areas of Barcelona (Spain). In addition, the approached drugs were also observed in the aquifer (Jurado et al., 2021).

Besides the individual risk of pharmaceuticals, the mixture of contaminants might also pose an unacceptable risk to aquatic habitats (Kumari and Kumar, 2022). But unfortunately, few studies approached the effects of NSAIDs toxicity in the aqueous environment (Scheurell et al., 2009; Maculewicz et al., 2022). In this sense, the toxicity of IBU after photodegradation was investigated using the algae *Chlorella* sp. with the determination of the toxicity of the obtained byproducts mixture, as well as the toxicity of the main photodegradation product 4-acetylbenzoic acid alone. No effect of the mixture on algae viability was observed in comparison to IBU, whereas 4-acetylbenzoic acid significantly affected *Chlorella* sp. viability, starting from 0.25 mM to concentrations above 0.5 mM that caused the death of all cells (Gong et al., 2021).

Moreover, chronic toxicity trials performed on *Oncorhynchus mykiss* evidenced cytological changes in the liver, kidney, and gills after 28 days of exposure to just 1 μg L^−1^ of DCF. When exposure reached concentration of 5 μg L^−1^, renal lesions were evident as well as drug accumulation in liver, kidneys, gills, and muscle. DCF also inhibited the growth of marine phytoplankton *Dunaliella tertiolecta* at 25 mg L^−1^ and above (DeLorenzo and Fleming, 2008). Approaching IBU ecotoxicity, it was reported generating growth stimulation of the cyanobacterium *Synechocystis* sp. for 5-day exposure to concentrations in the range of 1–1,000 μg L^−1^, but inhibition of the duckweed plant *Lemna minor* after 7 days (Pomati et al., 2004).

Even though NSAIDs are characterized by relatively low environmental stability, especially through biotransformation in aerobic conditions and photolysis occurring in surface waters, these compounds and metabolic residues are frequently detected in surface water, marine water, freshwater reservoirs, rivers and lakes, from concentration of ng L^−1^ up to μg L^−1^ (Świacka et al., 2020). Although physicochemical processes are commonly used in the remediation of NSAIDs, these processes need specific conditions for reaction, expensive operational costs, and may cause the formation of more toxic intermediate compounds (Sharma et al., 2020). Therefore, methods of green bioremediation processes are an attractive alternative for transformation of NSAIDs into low toxicity product and further mineralization.

## 4. Scope of research and bibliometric analysis

Search engines including Google Scholar, Web of Science, and Dimension were used to retrieve literature. Almost 32 research articles published between 2012 and 2023 (February) were employed for gathering relevant information. The primary search terms were “biodegradation and (diclofenac, ibuprofen, or paracetamol),” “bacteria removal and (diclofenac, ibuprofen, or paracetamol),” and “fungi removal and (diclofenac, ibuprofen or paracetamol).” In addition, a comprehensive bibliometric analysis was carried out using the international scientific database Scopus, aiming at mapping and understanding the interest and evolution of studies related to NSAIDs during the COVID-19 pandemic. To carry out this study, the methodology proposed by different authors was employed and adapted (Ucella-Filho et al., 2022; Lucas et al., 2023). In order to collect data relevant to the approached study topic, a search command was drawn up which included keywords pertinent to the focus of this review, and that were present in the titles or abstracts of articles. The following search formula was therefore used:: “TITLE (COVID-19 OR SARS-CoV-2 OR coronavirus OR COVID19) AND ABS (diclofenac OR ‘diclofenac potassium’ OR ‘paracetamol’ OR ‘acetaminophen’ OR ‘n-acetyl-p-aminophenol’ OR ‘ibuprofen’ OR ‘2-[4-(2-methylpropil)phenyl]propanoic acid)]’) OR TITLE (diclofenac OR ‘diclofenac potassium’ OR ‘paracetamol’ OR ‘acetaminophen’ OR ‘*n*-acetyl-p-aminophenol’ OR ‘ibuprofen’ OR ‘2-[4-(2-methylpropil)phenyl]propanoic acid)]’)) AND PUBYEAR >2012 AND PUBYEAR <2024.” The data collected was processed using the “biblioshiny” function in the “bibliometrix” package (Aria and Cuccurullo, 2017) of the R software (R Core Team, 2021), allowing us to obtain a structured and quantitative analysis of emerging trends in the field of NSAIDs research over the last 10 years (2013–2023).

Beyond the biodegradation process, several studies relating COVID-19 to drugs categorized as NSAIDs have received significant attention in the scientific community, increasing the number of articles aiming at a better understand of these drugs effects on the development of COVID-19. Therefore, justifying the environmental contamination by NSAIDs that might be present too in hospital, pre-clinical and clinical testing laboratories, as well as in universities and research institutes. In this context, it was carried out a comprehensive bibliometric analysis of articles mentioning relationships between NSAIDs and COVID-19, validating this information.

**Figure 2:**
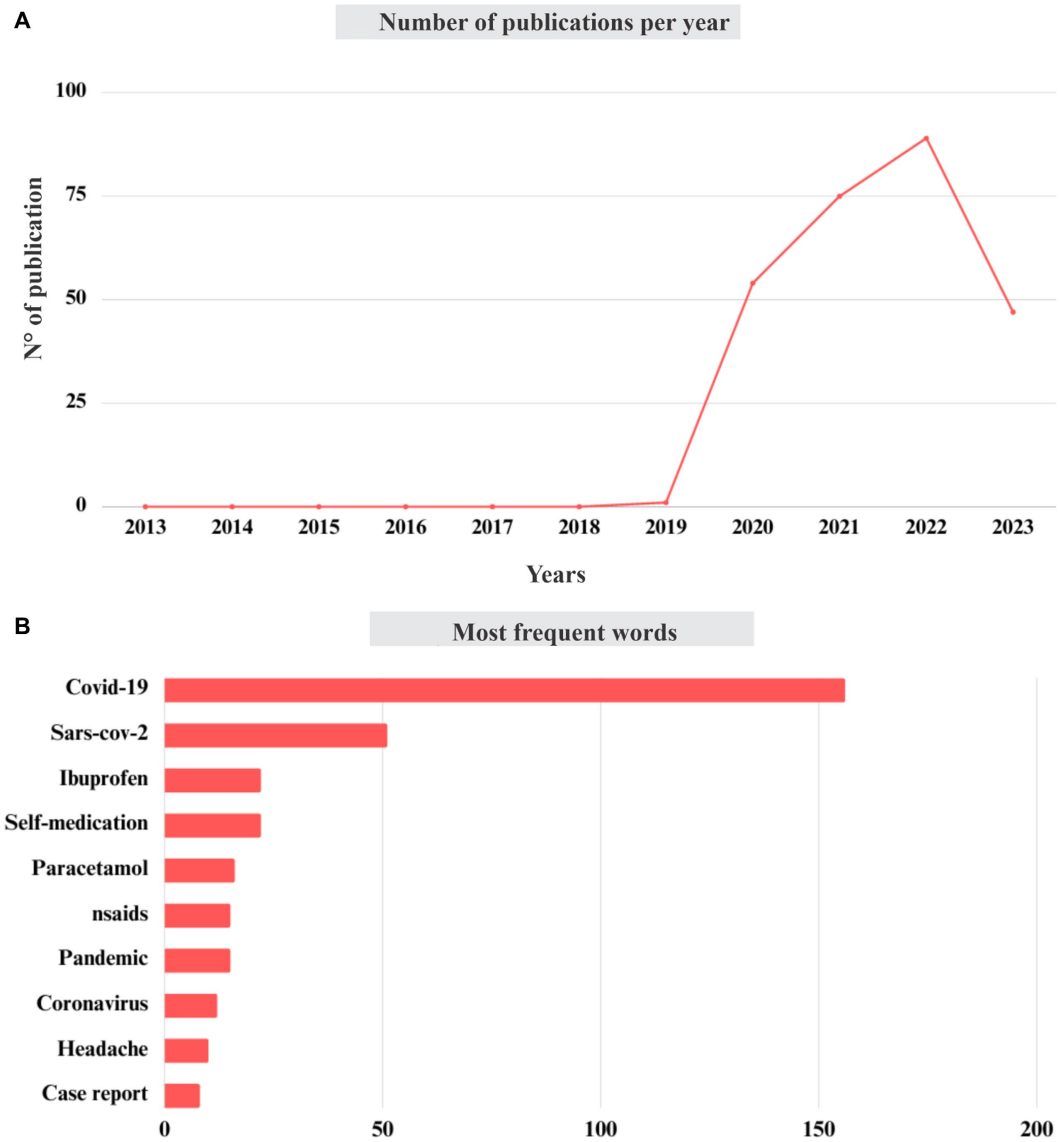
**(A)** Number of publications by year (2013–2023), and most frequent words **(B)** related to COVID-19 with NSAIDs.

A total of 344 articles addressing this current topic under analysis were identified, of which 76 were classified as review articles, book chapters, letters, and communications, whereas 268 were categorized as research articles, which served as the basis for the data presented in this discussion. As expected, there were no studies relating COVID-19 to NSAIDs in the period from 2013 to 2018. However, in 2019, with the clinical urgency for information and treatments of this new disease, the first studies were already described (Cheng et al., 2020). However, the highest number of publications was observed in the year of 2022 with 89 documents addressing this connection. Although there was a sharp decline (>50 articles) in the number of publications from 2023 onwards (Figure 2A). The continuous increase in publications over these years focusing on the use of NSAIDs in relation to the treatment of COVID-19 symptoms has become important, given the mutations and adaptations of this virus and their variants (Markov et al., 2023), as well the need for effective strategies for symptoms relieve and complications prevention.

This research also plays a key role in adapting clinical protocols, discovering innovative therapies, and advancing scientific knowledge to understand future pandemics. Among the highlighted words recorded in these papers and processed by the bibliometric method, IBU and PAR appear among the five most cited words, indicating their relevance in the context of COVID-19 (Figure 2B). Therefore, without going into the merits of whether concomitant treatment with NSAIDs can be harmful or safe in COVID-19 patients, the production and consumption of NSAIDs, such as IBU, PAR, and DCF, as well as the involvement in clinical and pre-clinical trials, are still considered high and necessary, especially in developed countries such as the United States, Spain, the United Kingdom, Italy, and other European countries (Austin et al., 2022). Thus, contributing to the detection of these substances (IBU, PAR, and DIC), in some cases, at concentrations above the legislative limit. Furthermore, it is also necessary to reflect on the biodegradation methods, which are intrinsically related to the extensive use of NSAIDs and studies of the pharmacological and epidemiological profile of COVID-19.

Therefore, means of removal and methods of (bio)remediation such as those presented here by fungi or bacteria must be continued for the development of applied bioremediation alternatives, in synergy with the production and evaluation studies (*in vitro* or *in vivo*) of these drugs. Aiming at limiting the impacts possibly generated by the release of the drugs into the environment.

As showed in Figures 2A,B, COVID-19 has dominated the global scientific focus since the beginning of 2020, and as such, researchers were dedicated to add their contributions to what is known and what can be done to combat COVID-19, in addition, studies involving NSAIDs are accelerated to support some approaches. Furthermore, the exhibited ability of the virus to mutate rapidly and access to public or private funding for COVID-19 research is still attractive (Brandt et al., 2022), consequently, the use of related substances, such as NSAIDs, added to the usual worldwide consumption of these substance will keep the consumption high for several years. Therefore, efficient methods for bioremediation process for DCF, IBU, and PAR will be important for understanding the impact of these drugs on the environment and environmentally exposed population.

## 5. Biodegradation of diclofenac

Diclofenac, [2-(2-(2,6-dichlorophenylamino)phenyl] acetic acid, is a common non-steroidal anti-inflammatory drug (NSAID) used as oral tablets or topical gel with an estimated global consumption of up to 940 t per year (Zhang et al., 2008), its physicochemical properties are presented in Table 1. After oral administration, 65 and 70% of DCF is excreted in urine and 20–30% in feces as parent drug or metabolites, thus this pharmaceutical undergoes almost complete biotransformation in the human body (Vieno and Sillanpää, 2014; Davis et al., 2020).

**Table 1:** Physicochemical properties of DCF, IBU, and PAR.

Parameter	DCF	IBU	PAR
Chemical structure	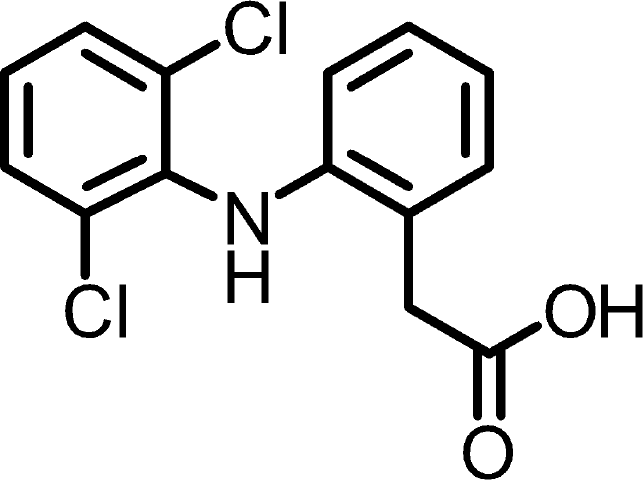	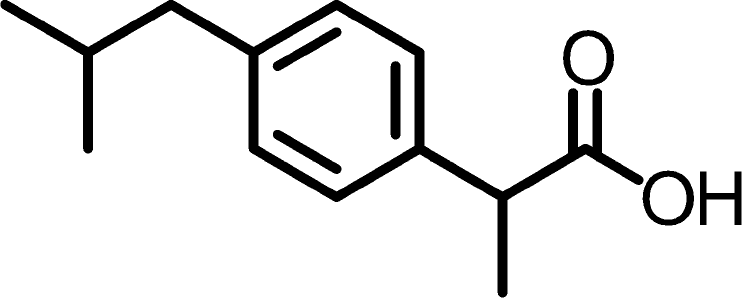	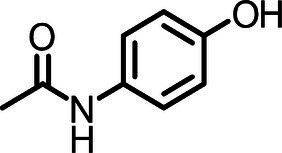
Chemical formula	C_14_H_10_C_l2_NO_2_	C_13_H_18_0_2_	C_8_H_9_NO_2_
CAS no	15307-86-5	15687-27-1	103-90-2
Water solubility	2.37 mg L^−1^	21 mg L^−1^	14 g L^−1^
pKa	4.15^a^	4.5^b^	9.38^c^
logKow	4.51^a^	2.48^b^	0.46^c^

^a^Zhang et al. (2008); ^b^Scheytt and Mersmann (2005); ^c^Ward and Alexander-Williams (1999).

Approaching the biodegradation by bacteria, strains isolated from a municipal wastewater treatment plant (WWTP) located in Northern Portugal (Ponte de Moreira, Portugal) were used for DCF biodegradation. *Brevibacterium* sp. D4 biodegraded 35% of 10 mg L^−1^ DCF as the sole carbon source, however the yield raised to 90% when periodic acetate addition as supplementary carbon source was employed (Table 2, entry 1; Bessa et al., 2017).

**Table 2:** Summary of DCF, IBU, and PAR biodegradation.

Ent.	Microorganism	Isolation place	Drug	Biodegradation rate (conditions)	Ref
1.	*Brevibacterium* sp. *D4*	From wastwater treatment plant (Ponte de Moreira, Maia—Portugal)	DCF	90% (10 mg L^−1^, at 25°C, 150 rpm by 30 days)	Bessa et al. (2017)
2.	*Labrys portucalensis* F11	From sediment of a polluted site in the northern Portugal	DCF	>99% (7.01 mg L^−1^ at 25°C, 130 rpm by 25 days)	Moreira et al. (2018)
3.	*Bacillus subtilis*	Provided by the Prodibio Company—Marseille, France	DCF	>99% (1,000 mg L^−1^, at 20°C, 100 rpm by 17 h)	Grandclément et al. (2020)
4.	*Rhodococcus ruber* IEGM 346	From Regional Specialized Collection of Alkanotrophic Microorganisms	DCF	>99% (1,000 mg L^−1^, at 28°C, 100 rpm by 6 days and 0.5% glucose).	Ivshina et al. (2019)
5.	*Klebsiella* sp. KSC	From livestock soil	DCF	90% (70,000 mg L^−1^, at 30°C, pH 7, 100 rpm by 72 h)	Stylianou et al. (2018)
6.	*Microbial consortia*	Native microbial soil	DCF	90% (1,000 mg L ^−1^, at 25°C, 120 rpm by 10 days)	Facey et al. (2018)
7.	*Ganoderma applanatum*	Collection in the Department of Pure and Applied Botany—Nigeria	DCF	61% (15 mg L^−1^, at 30°C, pH 4, 5 and 150 rpm by 72 h)	Bankole et al. (2020)
8.	*Laetiporus sulphureus*	Collection in the Department of Pure and Applied Botany, Federal University of Agriculture Abeokuta—Nigeria	DCF	73% (15 mg L^−1^, at 30°C, pH 4.5 and 150 rpm by 72 h)	Bankole et al. (2020)
9.	*Talaromyces gossypii*	Sewage sludge composite samples were collected from the WWTPs—Granada—Spain	DCF	84.6% (20.62 mg L^−1^ at 28°C by 72 h)	Conejo-Saucedo et al. (2021)
10.	*Syncephalastrum monosporum*	Sewage sludge composite samples were collected from the WWTPs—Granada—Spain	DCF	82% (20.62 mg L^−1^ at 28°C by 72 h)	Conejo-Saucedo et al. (2021)
11.	*Aspergillus tabacinus*	Sewage sludge composite samples were collected from the WWTPs—Granada—Spain	DCF	76% (20.62 mg L^−1^ at 28°C by 72 h)	Conejo-Saucedo et al. (2021)
12.	*Talaromyces verruculosus*	Sewage sludge composite samples were collected from the WWTPs—Granada—Spain	DCF	37% (20.62 mg L^−1^ at 28°C by 72 h)	Conejo-Saucedo et al. (2021)
13.	*Aspergillus terreus*	Sewage sludge composite samples were collected from the WWTPs—Granada—Spain	DCF	49,7% (20.62 mg L^−1^ at 28°C by 72 h)	Conejo-Saucedo et al. (2021)
14.	*Aspergillus cejpii*	Sewage sludge composite samples were collected from the WWTPs—Granada—Spain	DCF	14.6% (20.62 mg L^−1^ at 28°C by 72 h)	Conejo-Saucedo et al. (2021)
15.	Microbial consortium (*Alcaligenes faecalis, Staphylococcus aureus, Staphylococcus, haemolyticus, Proteus mirabilis*)	Isolated from the vicinity of a pharmaceutical manufacturing unit—India	DCF	45% (150 mg.L^−1^, at 25–30°C, pH 7 by 120 h)	Murshid and Dhakshinamoorthy (2019)
16.	*Penicillium oxalicum*	Isolated from a hydrocarbonpolluted pond in Motril—Granada	DCF sodium	99% (20.62 mg L^−1^, 24 h, 28°C)	Murshid and Dhakshinamoorthy (2019)
17.	*Rhizophagus irregularis*	Institute of Botany, Czech Academy of Science—Czechia	IBU	80% (0.5 mg L^−1^, 5–30°C, by 150 days,)	Hu et al. (2021)
18.	*Sphingopyxis granuli* RW412	River Elbe taken downstream of the Hamburg harbor-Germany	IBU	80% (800 mg L^−1^, 30°C, 200 rpm, 3 days)	Aguilar-Romero et al. (2021)
19.	*Bacillus thuringiensis*	Soil of the Chemical factory “Organika-Azot” in Jaworzno, Poland	IBU	46.56% (25 mg L^−1^, at 30°C,130 rpm by 20 days)	Marchlewicz et al. (2016)
20.	*Patulibacter* sp. Strain L11	Activated sludge collected from Beirolas WWTP—(Lisbon, Portugal)	IBU	92% (0.05 mg L^−1^, at 28°C, 110 rpm by 90 h)	Almeida et al. (2013a)
21.	*Gordonia amicalis* EU266486.1	Activated sludge Beirolas WWTP (Lisbon, Portugal)	IBU	26% (0.1 mg L^−1^, at 27°C, 110 rpm by 100 h)	Almeida et al. (2013b)
22.	*Acinetobacter bouvetti* JF681285	Activated sludge Beirolas WWTP (Lisbon, Portugal)	IBU	12.8% (0.1 mg L^−1^, at 27°C, 110 rpm by 100 h)	Almeida et al. (2013b)
23.	*Bacillus siamensis* DSI-1	Isolated from wastewater—GenBank MT 039503	IBU	50% (3.5 mg L^-1,^ at 30°C, pH 7, 165 rpm, by 18 h)	Chopra and Kumar (2022)
24.	*Microbacterium paraoxydans*	Isolated from pharmaceutical wastewater of East India Pharmaceutical Private Limited, Durgapur, West Bengal, India	IBU	92,01% (15 mg L^−1^, at 30°C, pH 7, 150 rpm, 0.3% yeast extract)	Show et al. (2023)
25.	*Patulibacter medicamentivorans*	Isolated from activated sludge collected from Beirolas WWTP (Lisbon, Portugal)	IBU	>99% (0.25 mg L^−1^, at 28°C, pH 7, 110 rpm by 7 days)	Salgado et al. (2020)
26.	*Nocardioides carbamazepini* sp. nov. CBZ_1T	Biofilm sample collected from a Pump and Treat system treating BTEX (benzene, toluene, ethyl-benzene, and xylenes) contaminated groundwater	IBU	70% (1.5 mg.L^−1^, at 27°C, 145 rpm by 7 days)	Benedek et al. (2022)
27.	*Sphingomonas wottichii* MPO218	Sewage sludge of Copero (EMASESA, dos Hermanas, Seville, Spain), Company Almirall (Barcelona Spain)	IBU	>75% (4.4 mM at 4.5 h, 30°C and pH 7)	Aulestia et al. (2021)
28.	*Sphingomonas* sp		IBU	>99% (500 mg L^−1^, 80 h, 37 ± 2°C)	Murdoch and Hay, 2013
29.	*Pseudoalteromonas* sp	GenBank accession number: KY583737	IBU	80%, (1.0 mg L^−1^, 20 ± 1°C, 150 rpm by 72 h)	Li et al. (2022)
30.	*Rhodococcus cerastii IEGM 1278*	Regional specialized Collection of Alkano-trophic Microorganisms (acronym IEGM, the wprdl Federation for Culture)	IBU	14.1% (100 mg L^−1^, at 28°C, 160 rpm by 7 days)	Ivshina et al. (2021)
31.	*R. cercidiphylli* IEGM 1184	Regional specialized Collection of Alkano-trophic Microorganisms (acronym IEGM, the wprdl Federation for Culture)	IBU	21.6% (100 mg L^−1^, at 28°C, 160 rpm by 7 days)	Ivshina et al. (2021)
32.	*R. erythropolis* IEGM 501	Regional specialized Collection of Alkano-trophic Microorganisms (acronym IEGM, the wprdl Federation for Culture)	IBU	18.6% (100 mg L^−1^, at 28°C, 160 rpm by 7 days)	Ivshina et al. (2021)
33.	*Paracoccus aminophilus* NR_042715.1	Activated sludge Beirolas WWTP (Lisbon, Portugal)	IBU	16.2% (0.1 mg L^−1^, at 27°C, 110 rpm by 100 h)	Almeida et al. (2013b)
34.	*Patulibacter americanus* NR_042369	Activated sludge Beirolas WWTP (Lisbon, Portugal)	IBU	35% (0.1 mg L^−1^, at 27°C, 110 rpm by 100 h)	Almeida et al. (2013b)
35.	*Pseudomonas stutzeri* CSW02	Sewage sludge was obtained from a Wastewater Treatment Plant (WWTP) in the city of Seville	PAR	100% (500 mg L^−1^, at 30 ± 1°C, 150 rpm by 4 h)	Vargas-Ordóñez et al. (2023)
36.	*Pseudomonas extremaustralis* CSW01	Sewage sludge was obtained from a Wastewater Treatment Plant (WWTP) in the city of Seville	PAR	100% (500 mg L^−1^, at 30 ± 1°C, 150 rpm, by 6 h)	Vargas-Ordóñez et al. (2023)
37.	*Pseudomonas* sp. PrS10	Sample wastewater collected from Cadila Pharmaceutical Limited, Gujarat, India	PAR	96.37% (3,000 mg L^−1^, at 30°C, 140 rpm by 7 days)	Poddar et al. (2022)
38	*Pseudomonas moorei* KB4	From the activated sludge from the wastewater treatment plant Klimzowiec (Chorzów, Poland)	PAR	99% (50 mg L^−1^, at 30°C, pH 7 by 1.5 h)	Żur et al. (2018b)
39.	*Pseudomonas aeruginosa*	Sludges from two Portuguese WWTPs	PAR	90% (50 mg L^−1^, at r.t, by 2 days)	Palma et al. (2018)
40.	*Pseudomonas* sp. f2	PAR-degrading aerobic aggregate	PAR	100% (2.000 mg L^−1^, at 30°C, 200 rpm by 70 h)	Zhang et al. (2013)
41.	*Pseudomonas* sp. fg-2	PAR-degrading aerobic aggregate	PAR	100% (2,500 mg L^−1^, 30°C, 200 rpm by 45 h)	Zhang et al. (2013)
42.	*Stenotrophomonas* sp. f1	PAR-degrading aerobic aggregate	PAR	100% (2,000 mg L^−1^, at 30°C, 200 rpm, by 16 h)	Zhang et al. (2013)
43.	*Pseudomonas aeruginosa strain* HJ1012	Isolated from stable microbial aggregate in a sequencing batch reactor	PAR	71.4% (2,200 mg L^−1^, at 30°C, pH 7 by 18 h)	Hu et al. (2013)
44.	*Aspergillus niger*	Wastewater samples were collected from the effluent generated by YEDCO factory situated in Sana’a City, Yemen	PAR	37% (2,000 mg L^−1^, at 25°C, pH 6.0 by 60 days)	Sana (2018)
45.	*Fusarium oxysporium*	Wastewater samples were collected from the effluent generated by YEDCO factory situated in Sana’a City, Yemen	PAR	26.1% (1,000 mg L^−1^, at 25°C, pH 6.0 by 60 days)	Sana (2018)
46.	Microbial consortium (*Bacillus cereus; Corynebacterium nuruki; Enterococcus faecium*)	A sample of aerobic activated sludge was collected from the oxidation ditch of Faro Northwest WWTP’s, Portugal.	PAR	> 90% (200 mg L^−1^, at 28°C, by 48 h)	Palma et al. (2021)
47.	*Bacillus drentensis estirpe* S1	The sewage samples used in this study were collected from waste water drain in Sonipat, Haryana, India	PAR	93% (300 mg L^−1^, at 40°C, pH 7, 165 rpm by 48 h)	Chopra and Kumar (2020)
48.	*M. yunnanensis* KGP04	Pharmaceutical industry wastewater and sludge samples were collected from VKIA (Vishwakarma Industrial Area), Jaipur	PAR	80% (1% w/v, at 25°C, pH 8, 200 rpm, by 6 h)	Sharma et al. (2020)
49.	*M. yunnanensis* TJPT4	Bacteria recovered from marine organisms, which were collected from the Catedral and Queijo Suiço marine caves, located in Sagres, Algarve, Portugal	PAR	>60% (15 mg L^−1^, at 28°C, 150 rpm, by 360 h)	Palma et al. (2022)
50.	*Pseudomonas* spp.	Sludge of a hospital WWTP (Pharmafilter, Delft, NL)	PAR	>99% [250 mgL^−1^ (reactor conditions 500 rpm, pH 7, airflow 30 mL/min, and 20 ± 1°C) at 10 days]	Rios-Miguel et al. (2022)

**Figure 3:**
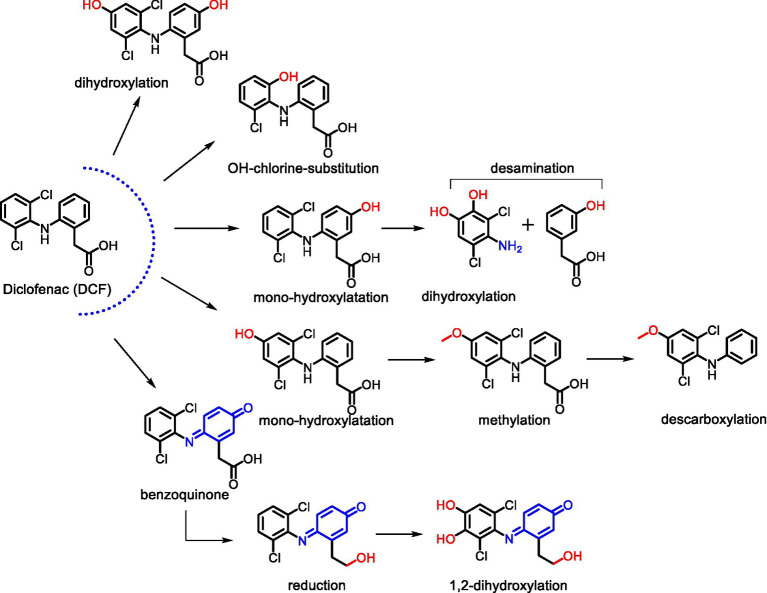
Schematic representation of DCF degradation by fungi or bacteria described in the literature (Bessa et al., 2017; Ivshina et al., 2019; Wojcieszyńska et al., 2023).

In another study, biodegradation of >99% DCF was reported by the bacterial strain *Labrys portucalensis* F11 (Table 2, entry 2) isolated from sediment of a polluted site in Portugal. Complete degradation was reached by co-metabolism with acetate after 6 days for 1.7 μM DCF, and after 25 days for 34 μM DCF both at 25°C and 130 rpm (Moreira et al., 2018). Furthermore, 12 metabolites were identified using UPLC-QTOF analyses. Including mono-and di-hydroxylated compounds resulted from hydroxylation at the chlorine-substituted site, decarboxylation, methylation of a hydroxyl group, and formation of benzoquinone imine species (Figure 3).

In addition, mono-hydroxylated DCF products were reported at biodegradation by *Bacillus subtilis* and *Brevibacillus laterosporus* strains provided by the Prodibio Company (Marseille, France). Complete removal was observed in biodegradation with 1 mg mL^−1^ DCF after 17 h of an experiment at 20°C, pH 7, and 100 rpm (Table 2, entry 3; Grandclément et al., 2020).

Recently, Ivshina et al. (2019) reported the ability of 104 *Rhodococcal* strains isolated from municipal wastewater and deposited at the Regional Specialized Collection of Alkanotrophic Microorganisms (IEGM, WDCM 768, http://www.iegmcol.ru) to biodegrade DCF. The selected strain *Rhodococcus ruber* IEGM 346 (Table 2, entry 4) completely biodegraded 50 mg mL DCF after 6 days at 28°C in pH 7 with glucose (0.5%). Analysis by gas chromatography–mass spectrometry (GC–MS) confirmed the C-N bond cleavage and aromatic ring opening of DCF structure, generating benzoquinone the imine, mono-and di-hydroxylated derivatives, which were the main biodegradation metabolites (Ivshina et al., 2019).

Some of these metabolites were also identified in the DCF biodegradation by *Klebsiella* sp. KSC isolated from livestock soil (Gen Bank, accession number KX500307). The best biodegradation results, 90%, was obtained at 70 mg L^−1^ DCF after 72 h at 30°C, pH 7, and 100 rpm (Table 2, entry 5). Furthermore, acute ecotoxicity assays with *Vibrio fischeri* were performed for the obtained biotransformation products, which were less toxic than the parent compound (Stylianou et al., 2018). In another study, up to 1.0 g L^−1^ DCF was fully biodegraded by a native microbial soil consortium obtained from 11 forest soil samples after 10 days at 25°C and 120 rpm (Table 2, entry 6; Facey et al., 2018).

Different bacterial strains previously isolated from a pharmaceutical manufacturing unit were also employed for DCF biodegradation. The consortium of bacteria composed of *Alcaligenes faecalis* (MG995024), *Staphylococcus aureus* (MG576208), *Staphylococcus haemolyticus* (MG995021), and *Proteus mirabilis* (MH021605) at pH 7 and temperature range of 25–30°C removed 45% DCF (Table 2, entry 7). Subsequently, at pH between 6 and 8 and 25–35°C, the consortium showed an efficiency increase to 55%. These results showed that consortia can be an interesting approach for the biodegradation of these micropollutants (Murshid and Dhakshinamoorthy, 2019).

Fungi species were also employed in the biodegradation process of DCF. *Ganoderma applanatum* and *Laetiporus sulphureus* deposited in the Microbial Collection of the Department of Pure and Applied Botany located at the Federal University of Agriculture Abeokuta (Nigeria) were explored. The removal of DCF reached 96% of 15 mg L^−1^ by the co-culture of both strains after 72 h at pH 4.5, 30°, and 150 rpm. It is important to note that the same strains, *G. applanatum* and *Laetiporus sulphureus*, exhibited reduced efficiencies of 61% (Table 2, entry 8) and 73% (Table 1, entry 9), respectively, when employed individually. Showing that the co-culture of fungi for biodegradation promoted synergistic effects (Bankole et al., 2020).

Fungal species isolated from sewage treatment plants at Granada (Spain) were also tested for DCF biodegradation. The consumption after 72 h at 28°C was assessed for six isolated strains (Table 2, entry 10–15). *Talaromyces gossypii* showed the maximum DCF removal of 85% (Table 1, entry 10), whereas *Syncephalastrum monosporum* presented 82% (Table 2, entry 11), and 76% removal was observed for *A. tabacinus* (Table 2, entry 12). In addition, *Aspergillus terreus*, *Talaromyces verruculosus*, and *Aspergillus cejpii* promoted lower biodegradation percentages of 50, 37, and 15% of DCF (Table 2, entries 13, 14, 15), respectively (Conejo-Saucedo et al., 2021).

The fungus *Penicillium oxalicum* previously isolated from a lake polluted by hydrocarbons in Motril (Spain) was also used for DCF biodegradation. Removal of 99% was observed after 24 h at 28°C, showing the interesting efficiency of this strain (Table 2, entry 16; Olicón-Hernández et al., 2019).

So, the literature shows that several microorganisms (fungi and bacterium) have been reported to biodegrade diclofenac with high rates of biodegradation through different metabolic pathways, including hydroxylation, dihydroxylation, decarboxylation, deschlorination, C-N bond cleavage, and aromatic ring opening. However, advances in the ecotoxicological study of metabolites of DCF are necessary.

## 6. Biodegradation of ibuprofen

Ibuprofen, ((2rs)-2-[4-(2-methylpropil)phenyl]propanoic acid), is a NSAIDs used in the form of tablet, capsule, oral suspension, granule, suppository, cream, gel, drop, and injection. Its physicochemical properties are described in Table 1. Approximately 80% of the IBU dose is absorbed in the gastrointestinal tract when administered orally. Then, its biotransformation occurs in the liver generating glucuronide metabolites that are excreted in the urine, in which less than 1% of the dose is excreted in untransformed form (Davies, 1998).

Microbial degradation of IBU has received attention from the worldwide scientific community due to its extensive consumption (Rastogi et al., 2021). The development of IBU was a result from the search for safe drugs in the middle of the 21st century, entering the English market in 1967 and in America in 1974, thus this was the first propionic acid to be used in the United States with non-steroidal anti-inflammatory therapeutic properties. In a comparison with other treatment options, IBU has weak anti-inflammatory activity, but the adverse effects are low, resulting in frequent use for pain and fever relief (Kantor, 1984; Ren et al., 2021).

Although different biocatalysts have been presented for IBU biodegradation, the search for new strains with unique properties continues using different perspectives aims at new information and insights for applied bioremediation.

The use of fungal strains in the biodegradation process of IBU is understudied, but some studies were described. In this regard, IBU-mineralizing bacteria isolated from sediments of the Elbe River taken downstream of the Hamburg harbor (Germany) were explored. These strains were characterized and tested for IBU remediation in different media. *Sphingopyxis granuli* (RW412) biodegraded 80% of 0.08 mg g^−1^ IBU in 3 days at 30°C and 200 rpm (Table 2, entry 17). These results indicated that RW412 bioaugmentation in a biopurification system can improve the dissipation rates of this active ingredient (Aguilar-Romero et al., 2021).

Wastewater was explored for strains isolation by different authors. Such as in the biodegradation of IBU by *Patulibacter* sp. L11 isolated from activated sludge collected from Beirolas WWTPs (Lisbon, Portugal), which was performed at different concentrations (1,000, 250, and 50 μg L^−1^). Experiments at 28°C and 110 rpm resulted in 62% biodegradation of 50 μg L^−1^ initial concentration using M9 medium with yeast extract and tryptone (Table 2, entry 18). Moreover, 92% biodegradation was observed using OD2-medium (Almeida et al., 2013c).

Other strains obtained from activated sludge of Beirolas WWTPs were also explored for IBU biodegradation. The biodegradation reactions were performed at 27°C and 110 rpm for 100 h with an initial IBU concentration of 100 μg L^−1^. The species with the highest percentage of biodegradation was *Patulibacter americanus* NR_042369 with 35%, followed by *Gordonia amicalis* EU266486.1 with 26%, *Paracoccus aminophilus* NR_042715.1 with 16%, and *Acinetobacter bouvetti* (JF681285) with 13% biodegradation (Table 2, entry 19–20; Almeida et al., 2013b).

Also from the WWTP located in Beirola, *Patulibacter medicamentivorans* I11^T^ was explored at IBU biodegradation (7 days, 28°C, pH 7.0, 110 rpm) with focus on metabolite identification in mineral medium supplemented with tryptone and yeast extract (Table 2, entry 21; Almeida et al., 2013a). GC–MS and LC–MS/MS analyses revealed 22 byproducts and different biodegradation pathways were proposed based on hydroxylation reactions followed by the production of carboxylic acids and ring cleavage. Toxicity to different species and biodegradability prediction were performed using EPI Suite software, and it was concluded that compounds with increased hydrophilic character and reduced toxic effects were obtained (Almeida et al., 2013a; Salgado et al., 2020).

Active microbial cultures was also collected from treatment plants in India for isolation of bacterial IBU-degraders by enrichment culture technique. The identified strain *Bacillus siamensis* DSI-1 was assessed in an effluent sample supplemented with IBU. After 18 h, 50% biodegradation was determined and total removal was achieved in 15 days. The kinetic analysis was performed in batch experiments and optimum conditions were 30°C, pH 7, 165 rpm, and 3.5 mg L^−1^ of IBU initial concentration, reaching 86% of biodegradation and showing that optimization strategies should be employed for the obtention of higher process yields (Table 2, entry 22; Chopra and Kumar, 2022).

*Microbacterium paraoxydans* Genbank OL614700 and OL614701 (Table 2, entry 23) from an industrial wastewater facility at Durgapur, also at India, were screened in mineral salt medium and employed for IBU biodegradation. An interesting process optimization by central composite design was performed and a maximum biodegradation of 92% was obtained at pH 7, inoculum of 0.1 OD_600_, 150 rpm, 30°C, and 0.3% yeast extract. It is important to note that the most significant factor for biodegradation were yeast extract content, showing the importance of co-metabolism for enhanced efficiency (Show et al., 2023).

In an omics approach, the use of metagenomic, metatranscriptomic, and metabolic analyses indicated that *Nocardioides carbamazepini* sp. nov. CBZ_1T was a new strain with an interesting ability to biodegrade pharmaceutics, emphasizing carbamazepine and IBU. This biocatalyst presented 70% biodegradation of 1.5 mg.L^−1^ IBU after 7 days in a medium supplemented with glucose (Table 2, entry 24). Therefore, a new strategy was employed with metagenome binning that resulted in a specific biocatalyst of interest, showing a new perspective for biodegradation studies (Benedek et al., 2022).

The enzymatic and genetic aspect of IBU biodegradation was also approached in the biodegradation of IBU by *Sphingomonas* sp. Ibu-2, which was isolated from a sewage treatment plant at Ithaca, United States (Table 2, entry 25). This strain received attention due to its ability to break IBU acid chain to the corresponding catechol. Seven genes (ipfABDEFHI) were identified in the fosmid pFOS3G7 obtained from the chromosomal library of *Sphingomonas* sp. Ibu-2, which encoded the IBU and phenylacetate deacylation activity subsequently expressed in *E. coli*. These results expanded the knowledge about the genetic and enzymatic aspects of IBU biodegradation, enabling further process efficiency increase (Murdoch and Hay, 2013).

Recently, *Sphingomonas wittichii* MPO218 biodegraded IBU as the sole carbon source. Further exploration showed that this strain genome consisted of a circular chromosome and two circular plasmids. The plasmid pIBU218 presented a region with 100% identity with an IBU catabolic gene cluster previously described (Table 2, entry 26). Moreover, this plasmid was conjugative, enabling the horizontal transfer of the IBU consumption ability to *Sphingopyxis granuli* TFA, showing that gene transfer plays an important role in bacterial communities at biodegradation (Aulestia et al., 2021).

Industrial environments were also explored for the obtention of interesting biocatalysts. For instance, the Gram-positive *Bacillus thuringiensis* B1 was isolated from the soil of a chemical factory in Jaworzno (Poland). This strain biodegraded 20 mg L^−1^ of IBU in 6 days at 30°C using glucose as carbon source (Table 2, entry 27), showing its potential for biodegradation (Marchlewicz et al., 2016).

Marine aphotic environment was also explored for IBU biodegradation in the search for new biocatalysts, including *Pseudoalteromonas* sp. GCY isolated from sediment. Experiments with 1 mg.L^−1^ IBU at 20°C and 150 rpm for 72 h reached about 94% efficiency and five biodegradation products were suggested (Table 2, entry 28). Furthermore, an extensive approach showed that both abiotic and biotic degradation mechanisms occurred by the conversion of IBU by extracellular Reactive Oxygen Species to 4-ethylresorcinol, which was later converted by different intracellular enzymes. These results showed the importance of unifying different experimental data to reach an expanded overview of a biodegradation process (Li et al., 2022).

Extensive screenings of specific groups of strains were employed by some groups for xenobiotics biodegradation. In this regard, 100 actinobacteria strains were assessed, and *Rhodococcus cerastii* IEGM 1278 was selected by the total consumption of 100 mg.L^−1^ IBU after 144 h in the presence of n-hexadecane as alternative carbon source. IBU promoted the transition from the single to the multicellular form of *R. cerastii* IEGM 1278, which was accompanied by pronounced morphological changes in cell shape, size, and surface roughness. Moreover, the six obtained byproducts resulted from hydroxylation and decarboxylation reactions presented higher phytotoxicity than IBU. Thus, indicating a relevant environmental issue during this biodegradation process. In the case of medium supplemented with 100 mg L^−1^ IBU and 0.1% glycerol, *Rhodococcus cerastii* IEGM 1278, *R. cercidiphylli* IEGM 1184, and *R. erythropolis* IEGM 501 promoted 14, 22, and 19% biodegradation, respectively, after 7 days (Table 2, entry 29–31; Ivshina et al., 2021).

In an applied bioremediation approach, the effects of colonization of the arbuscular mycorrhizal fungus (AMF) *Rhizophagus irregularis* BEG140 on the growth and treatment of wetland plants (*Glyceria maxima*) under selective pressure by the presence of IBU were investigated in constructed wetlands. Two treatments were adopted in 52 days of reaction, sterilized inoculum and fungal inoculum, in which the removal efficiencies of 0.5 mg L^−1^ IBU were 74–87 and 88–93% (Table 2, entry 32), respectively. Therefore, bioaugmentation promoted a slight increase in IBU biodegradation rates (Hu et al., 2021).

Some studies reported the isolation and identification of IBU-biodegrading strains of bacteria, also approaching biodegradation pathways. However, few research groups have been performing integrative omics approaches, including genomic and metabolomics assessments aiming at deeper knowledge about the biodegradation of IBU, which is an extensively employed pharmaceutical and micropollutant. Furthermore, the potential of fungi for biodegradation of this compound is unknown, since an adequate number of studies are required for the obtention of wider conclusions.

**Figure 4:**
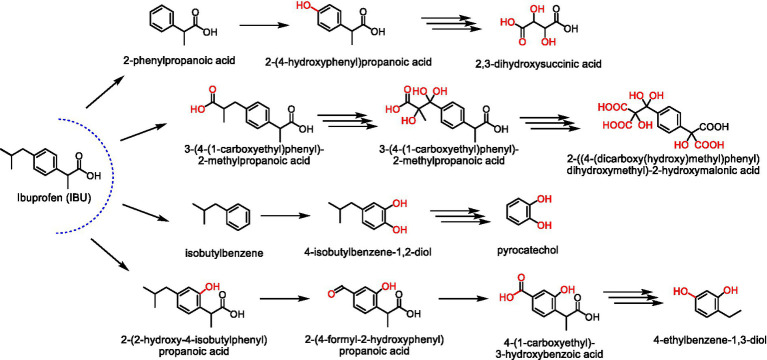
Some metabolites of IBU degradation from fungi or bacteria are described in the literature (Salgado et al., 2020; Li et al., 2022).

Figure 4 shows IBU different biodegradation pathways, including the formation of isobutylbenzene, 2-(2-hydroxy-4-isobutylphenyl)propanoic acid, and 3-(4-(1-carboxyethyl)phenyl)-2-methylpropanoic acid, which were produced in the first step of degradation. Furthermore, the formation of 2-(4-formyl-2-hydroxyphenyl) propanoic acid can be observed, as well as other byproducts that results from decarboxylation and dehydrogenation reactions (Salgado et al., 2020; Li et al., 2022).

## 7. Biodegradation of paracetamol

Paracetamol or acetaminophen (*N*-acetyl-*para*-aminophenol, PAR) is an analgesic and antipyretic drug available in the form of tablet and oral suspension, its physicochemical properties are presented in Table 1. This pharmaceutical is administered orally and 90% of the dose is metabolized in the liver producing 5–15% of the hydroxylated product *N*-acetyl-*p*-benzoquinone imine by the enzyme cytochrome P-450, and the elimination of 5% through the kidneys in unchanged form (Wojcieszyńska et al., 2022).

Due to its large-scale use, this drug can be found in different environments, especially in water bodies. Regarding this, it is noteworthy that PAR is bioaccumulative in aquatic organisms and may induce reproductive and neurotoxic disorders (Vargas-Ordóñez et al., 2023). Therefore, this compound was considered an emerging pollutant that requires environmentally safe strategies for its removal.

**Figure 5:**
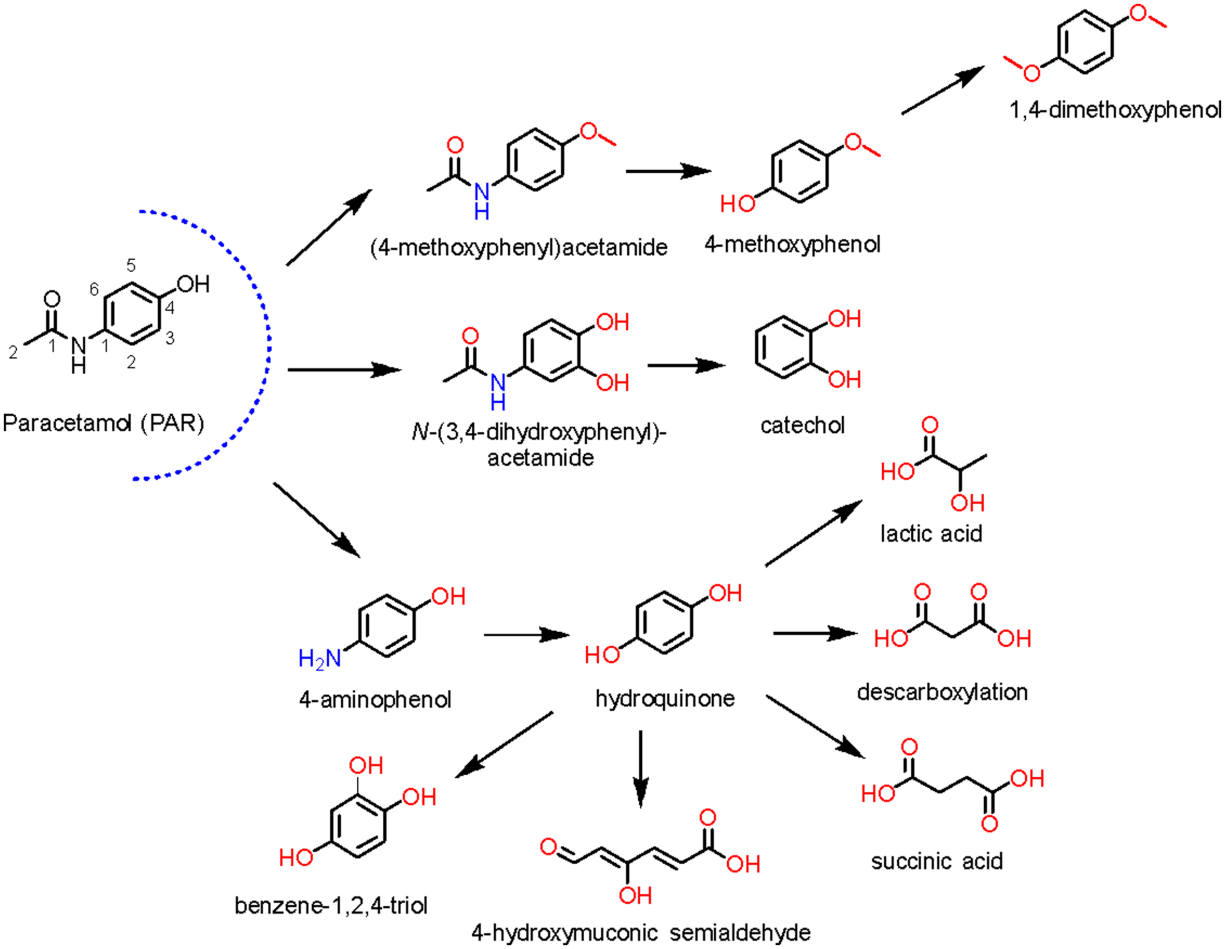
Some PAR metabolites from fungi or bacteria are described in the literature (Żur et al., 2018a; Rios-Miguel et al., 2022).

Microorganisms, mainly bacteria, have been reported as efficient biocatalysts for PAR biodegradation in different environmental matrices, in which the most frequently detected products are 4-aminophenol, hydroquinone, 2-hexenoic acid, succinic acid, malonic acid, oxalic acid, formic acid, nitrate, and nitrite. However, the first step in PAR biodegradation is the cleavage of the amide bond by an amidase to produce 4-aminophenol and acetate (Figure 5; Hu et al., 2013).

The screening of bacterial strains has been an important tool for PAR biodegradation. For instance, 17 bacterial strains were isolated from the sewage of a Wastewater Treatment Plant (WWTP) in Seville (Spain), but only two of them (*Pseudomonas stutzeri* CSW02 and *Pseudomonas extremoustralis* CSW01) biodegraded high concentrations of PAR as sole carbon and energy source. The process reached 100% degradation at 500 mg L^−1^ PAR, 150 rpm, and 30°C for 6 h, showing the high efficiency of these strains (Table 2, entry 33 and 34, respectively; Vargas-Ordóñez et al., 2023).

In another study, several bacterial strains were isolated from wastewater at a pharmaceutical company in Gujarat (India). Recently, tests with *Pseudomonas* sp. PrS10 showed high biodegradation efficiency with removal of 96% of 3 g L^−1^ PAR at 30°C and 140 rpm for 7 days of reaction (Table 2, entry 35). Furthermore, experiments indicated that both cell surface absorption and internalization of PAR were relevant processes (Poddar et al., 2022).

Wastewater treatment plants were the most explored source of biocatalysts for PAR biodegradation. Thus, six new bacterial strains isolated from activated sludge at the Klimzowiec wastewater treatment plant (Chorzów, Poland) removed PAR. *Pseudomonas moorei* KB4 was the most efficient strain in the initial concentration of 50 mg L^−1^ PAR at 30°C and pH 7.0 for 1.5 h (Table 2, entry 36). In addition, the biodegradation products *p*-aminophenol and hydroquinone were identified (Żur et al., 2018a), then, the hydroquinone aromatic ring might have been cleaved to 4-hydroxymuconic semialdehyde (Figure 5). Furthermore, this study showed that the concentrations of aromatic metabolites (phenol, 4-hydroxybenzoate, 4-chlorophenol, and 2-chlorophenol) were observed for a longer period than PAR, whereas aminophenol fast disappearing was determined. Indicating that these substrates may compete with the same enzymes, a phenomenon frequently observed during the degradation of aromatic compounds (Guzik et al., 2014).

Bacterial communities isolated from sludge of two Portuguese WWTPs were explored for PAR biodegradation under different media and oxygen conditions. Aerobic bacteria completely removed PAR in wastewater at a concentration of 50 mg L^−1^ after 2 days of incubation with aeration, and the metabolites 4-aminophenol and hydroquinone were identified as PAR degradation products. These results revealed that *Pseudomonas*, initially in 0.1%, reached the final relative abundance of 21.2% during the biodegradation process, confirming previous works reporting strains of this genus as paracetamol decomposers. Besides, the genera *Flavobacterium*, *Dokdonella,* and *Methylophilus* also deserve to be highlighted, since the initial relative abundances of 1.7%, 1.5 and 0.0% (not detected) in the inoculum reached 6.9, and 3.8 and 3.8% after incubation, respectively. Indicating the putative role of these genera in paracetamol biodegradation (Table 2, entry 37; Palma et al., 2018).

Still approaching the pseudomonas genus, three bacterial strains (*Pseudomonas* sp. f2, *Pseudomonas* sp. fg-2, and *Stenotrophomonas* sp. f1) were isolated from a PAR degrading aerobic aggregate using this drug as a unique source of energy. Complete biodegradation was observed for initial concentrations of 2.000 mg L^−1^ PAR after 70 h, 2.500 mg L^−1^ after 45 h, and 2.000 mg L^−1^ after 16 h at 30°C, showing the high efficiency and potential of these strains (Table 2, entry 38–40). Furthermore, the metabolites 4-aminophenol and hydroquinone were detected (Zhang et al., 2013).

The species *Pseudomonas aeruginosa* HJ1012 isolated from a stable microbial aggregate in a sequential batch reactor was also employed for PAR removal. Complete biodegradation of this active ingredient was obtained after 18 h at pH 7.0 and 30°C with an initial concentration of 2.200 mg L^−1^ (Table 2, entry 41). The consumption of PAR by *Pseudomonas aeruginosa* HJ1012 generated 71.4% of CO_2_, showing that consumption occurred *via* mineralization (Hu et al., 2013).

Recently bacterial strains obtained from an oxidation ditch of a WWTP in Faro Norwest (Portugal) consumed PAR as carbon source. Strains were selected and *Bacillus cereus*, *Corynebacterium nuruki* and *Enterococcus faecium* removed 97 ± 4, 97 ± 6, and 87% PAR, respectively, at an initial concentration of 200 mg L^−1^ after 48 h at 28°C (Table 2, entry 42). Furthermore, the metabolites 4-aminophenol, hydroquinone, and 2-hexenoic acid were identified in this process, bringing new insights into the environmental fate of this drug (Palma et al., 2021).

In another study, *Bacillus drentensis* S1 obtained from sewage of a wastewater drainage in Sonipat (India) was employed in an experimental design in a 20 L batch reactor resulting in an increase from 28 to 93% of PAR removal in the optimized experimental conditions of pH 7.0, 40°C, and 165 rpm and PAR initial concentration of 300 mg L^−1^, showing that different factors can affect this biodegradation process (Table 2, entry 43; Chopra and Kumar, 2020).

Pharmaceutical industrial wastewater was also explored for PAR biodegradation. *Micrococcus yunnanensis* KGP04 was isolated for PAR biodegradation processes from sludge collected at the Vishwakarma industrial area (India). Removal of 43% was achieved in the initial screening and, after applying a Taguchi L8 experimental design for optimization, a biodegradation of 83% was obtained in optimized conditions [dextrose-0.15%, peptone 0.1%, inoculum size 4% (w v^−1^), rpm 200, pH 8 at 25°C] after 6 h (Table 2, entry 44). Moreover, the biodegradation products were explored using nuclear magnetic resonance and Q-Tof spectrometry, aiming at expanding the knowledge about the fate of this drug in the environment (Sharma et al., 2020).

Another strain from the same species was employed in another PAR biodegradation study. *Micrococcus yunnanensis* strain TJPT4 was isolated from consortia of marine organisms collected at Catedral and Queijo Suijo marine caves (Sagres, Portugal). This strain showed high biodegradation capacity with 93% removal of 15 mg L^−1^ PAR after 360 h (Table 2, entry 45), also consuming the obtained metabolites. Therefore, promoting a cleaner residual medium (Palma et al., 2022).

A diverse microbial community obtained from a hospital WWTP enriched under low PAR concentrations in a membrane bioreactor was also approached, and two bacterial strains were isolated. A fast-growing *Pseudomonas* sp. that biodegraded 200 mg L^−1^ PAR in approximately 10 h producing 4-aminophenol, and a slow-growing *Pseudomonas* sp. that degraded PAR without obvious intermediates in more than 90 days were obtained. In addition, the metabolites 4-aminophenol, hydroquinone, hydroxyquinol, 4-hydroxymuconic semialdehyde, 2,5-dihydroxy-6-oxo-2,4-hexadienoic acid, and 3-hydroxy-2,4-hexadienedioic acid were identified as biodegradation products of PAR, expanding the available information about the biodegradation pathways of these compounds (Figure 5; Rios-Miguel et al., 2022).

Fungi strains were also employed for PAR biodegradation. *Aspergillus niger* and *Fusarium oxysporium* isolated from wastewater of a pharmaceutical industry located in Sana’a (Yemen) were assessed. The optimized conditions were 25°C and pH 6.0 for both species with 37% biodegradation for *A. niger* with an initial concentration of 2000 mg L^−1^ and 26% for *F. oxysporium* with an initial concentration of 1,000 mg L^−1^ after 60 days of reaction, indicating that fungi strains can also be employed as biodegradation agents in PAR biodegradation processes (Table 2, entry 46 and 47; Sana, 2018).

Therefore, PAR can be degraded by different microorganisms, although bacteria were better explored than fungi for this purpose. Furthermore, the biodegradation of the obtained aromatic products includes hydroxylation reactions and cleavage of the aromatic ring, in general catalyzed by dioxygenases and monooxygenases. The main intermediates such as catechol, hydroquinone, 4-methoxyphenol, succinic, and lactic acid are formed as a result of these enzymatic hydroxylations (Rios-Miguel et al., 2022).

Many research groups focused their studies on optimizing the biodegradation process by different parameters, such as pH, temperature, oxygen availability, and nutrient supply, in interesting experiments that focused on the understanding how environmental factors influence efficiency. Regarding this, the obtained conditions were usually specific for each employed biocatalysts, but in general, extreme conditions jeopardized the process consumption rate. Furthermore, some results indicated that ideal conditions for strain growth and development can be an interesting starting point in terms of pH, temperature and oxygen supply, although the use of nutrient rich media or xenobiotics as sole carbon source can depend on the employed biocatalyst, since this ability to biodegrading these compounds as unique carbon source can be absent and other approaches should be employed for increased performance (Żur et al., 2018a).

In this context, tools from molecular biology should also be employed for obtaining increased efficiency, including characterization of microbial communities and identification of genus and species involved in high efficiency processes, as well as their relationships. Furthermore, omics approaches are necessary for a better comprehension of the genetic, enzymatic, and metabolic aspects involved in these reactions, since a comprehensive approach is necessary for the obtention of a process overview. Thus, these strategies could be employed in integration with previous studies in the development of applied technologies, especially for areas with increased NSAIDs at wastewater, as can occur at the surroundings of hospitals, clinics, and industrial plants.

## 8. Conclusion and future directions

Microbial degradation processes are a great alternative for the biodegradation of NSAIDs, especially using selected strains isolated from contaminated environments. Moreover, extensive studies about biodegradation pathways are required for the determination of the fate of these compounds, including the knowledge about enzymes and genes involved in these processes.

An increase in the amount of non-steroidal anti-inflammatory drugs, especially DCF, IBU, and PAR, present in domestic and hospital sewers was expected from the consumption of the population in the treatment of COVID-19-driven infections, resulting in aggravated environmental impact. Moreover, the consequences of NSAIDs should not be considered individually for each active ingredient, since complex mixtures and combinations with other xenobiotics can greatly enhance their impacts on nature and human health.

The growth of microbial degradation studies applied to NSAIDs mostly conducted with bacteria is remarkable, but the ecotoxicological consequences of biodegradation products are still unknown. Therefore, some topics require extensive advances, such as systematic monitoring of NSAIDs and its biodegradation products at *in vivo* models. Furthermore, studies addressing the effect of biodegradation products with mixtures of other active ingredients related to the treatment of different diseases, including COVID-19, are essential. Also, assessment on cost-performance and energetic efficiency of different degradation methods should be addressed.

It is important to emphasize the use of molecular biology for characterization of microbial communities integrated with omics tools for revealing the genetic, enzymatic and metabolic aspects behind these processes are desired, and should be in the mind of research groups. In addition to the selection and use of highly efficient strains for the development of applied technologies.

Therefore, the development of alternatives to minimize the increasing pollution promoted by the incorrect disposal, human excretion, or production activity of NSAIDs and related compounds will be essential for maintaining a healthy ecosystem and life.

## Author contributions

BF, DF, SB, AF, FH, and JU-F: methodology, validation, investigation, data curation, and writing—original draft. WB, RL, and IF: conceptualization, supervision, writing—review and editing, and methodology.
